# Factors associated with multiple transitions in care during the end of life following enrollment in a comprehensive palliative care program

**DOI:** 10.1186/1472-684X-5-4

**Published:** 2006-05-30

**Authors:** Beverley Lawson, Frederick I Burge, Patrick Critchley, Paul McIntyre

**Affiliations:** 1 Department of Family Medicine, Dalhousie University, Halifax, NS, Canada; 2 Active staff, Timmins and District Hospital, Timmins, ON, Canada; 3 Division of Palliative Medicine, Dalhousie University/Capital District Health Authority, Halifax, NS, Canada

## Abstract

**Background:**

Patients often experience changes or transitions in where and by whom they are cared for at the end of life. These cause stress for both patients and families. Although not all transitions during the end of life can be avoided, advance identification of those who could potentially experience numerous transitions may allow providers and caregivers to anticipate the problem and consider strategies to minimize their occurrence. This study examines the relationship between patient characteristics and the total number of transitions experienced by the patient from the date of admission to a palliative care program (PCP) to death and during final weeks of life.

**Methods:**

Subjects included all adults registered with the PCP in Halifax, Nova Scotia, Canada between 1998 and 2002 and who had died during that period. Data was extracted from the regional PCP database and linked to census information. Transitions were defined as either: 1) a change in location of where the patient was cared for; or 2) a change in which service (specialist groupings, primary care, etc) provided care. Descriptive statistics were calculated plus rate ratios for the association between patient characteristics and total number of transitions.

**Results:**

In total, 3972 patients made 5903 transitions during the study period. Although 28% experienced no transitions, over 40% experienced one and 6.3% five or more. At least one transition was made by 47% during the last four weeks of life. Adjusted results suggest women, the elderly and more recent death are associated with experiencing fewer transitions. Multiple transitions were associated with a hospital death and a cancer diagnosis. During the last month of life, age was no longer associated with the total number of transitions, cancer patients were found to experience a similar number or fewer transitions than patients with a non-cancer diagnosis and pain and symptom control become a significant factor associated with a greater number of transitions.

**Conclusion:**

Our data suggest there is some variation in the number of transitions associated with the demographics and diagnoses of patients. Associations with gender and age require further exploration as does the contribution of caregiver supports and symptom issues.

## Background

During the end of life patients often experience changes or transitions in where and by whom they are cared for. These situations may result in increased anxiety for both the patient and their caregivers [1]. Such transitions may involve a change in hospital wards, a move from hospital to home or long-term care facility or a change in health care provider. With each transition there is a need for planned and integrated transfer of patient information, both medical and personal, the reiteration of patient preferences and the renegotiation of care goals. Transitions also require the patient and their caregivers to create new or redefined relationships with an expanding set of health care providers, establish new communication channels and form new trusts [2]. Physical aspects of a transition also need to be addressed and implemented in a timely and coordinated matter in order to minimize the effect on the patient and their caregiver's experience of care. These include the provision of hospital equipment in the home or advanced planning for admission/transfer to an alternative ward or institution.

At the same time we acknowledge that transitions may also be quite welcomed by patients and families. When the stress of symptoms or caregiving situations is too much at home, a move to hospital may be a relief to all. The same may be true of a transition of service provider such that patients may see the most competent professionals needed are finally involved in their care.

Nevertheless, in our modern health care systems we must ensure that care that moves patients across care settings and across clinical programs is as integrated and as smooth as possible. Although not all transitions during the end of life can be avoided, reducing the number of unnecessary changes in settings and providers may alleviate some hazards associated with transitions and provide the patient and their caregiver with a better care experience and quality of life during the end of life.

The database maintained by the Palliative Care Program (PCP) at the Queen Elizabeth II Health Sciences Center (QEII) in Halifax, Nova Scotia, Canada provides us with a relatively unique opportunity to track how often and when transitions are experienced by patients admitted to the program in addition to the provision of some basic patient demographic information. The PCP is comprised of an in-patient acute care unit, an in-hospital consultation service and offers home consultation services and an oncology outpatient clinic consultation service. Team members include health care professionals such as physicians, nurses and pharmacists in addition to social workers, spiritual care providers and volunteers. Since opening in 1988, the PCP has experienced major gains in the proportion of advanced cancer patients within the Halifax Regional Municipality (population approximately 350,000) being admitted into the program, increasing from 21% of all those who died of cancer in 1988 to 62% by 1997 [3,4]. Once admitted to the PCP, changes or transitions in location and care provided to patients are documented on the program's electronic database [5].

By looking at the relationship between number of transitions and patient characteristics we hope to identify patient predictors of multiple transitions. Advance identification of those who could potentially experience numerous transitions will allow providers and caregivers to anticipate the problem and consider strategies to minimize their occurrence. Advance identification will also allow the patient and caregiver to prepare for these transitions, both physically and emotionally, before they occur.

In a companion paper we described the distribution of transitions in care experienced by palliative care patients during the time subsequent to admission to a comprehensive palliative care program [6]. In this paper we describe the relationship between patient characteristics and the total number of transitions experienced by the patient from the date of admission to the PCP to death and during the final two and four weeks of life.

## Methods

This study represents a further investigation of transitions experienced by all patients registered in a comprehensive palliative care program (PCP) at the Queen Elizabeth II Health Services Center (QEII) in Halifax, Nova Scotia, Canada between January 1, 1998 and December 31, 2002 with a recorded date of death on or prior to December 31, 2002. Details of the initial investigation describing the distribution of transitions in care among these patients have been previously published [6].

The research ethics board of the Nova Scotia Capital District Health Authority, Halifax, Nova Scotia provided ethical approval for this research. Personal identifying information was not included on the records used for this project. All patient information available was provided anonymously to preserve confidentiality.

### Data

Data for this study were obtained from the QEII PCP database, augmented with aggregate Statistics Canada Census (2001) information. During the five year study period, 4434 patients were admitted to the program; 90% with a cancer diagnosis. The number of patients admitted to the program each year remained relatively stable (mean, 887; standard deviation [SD] 38). Information contained within the PCP database is considered to be valid and reliable. Data are entered by a trained entry clerk who is dedicated to ensure the completeness and accuracy of all information. The data entry program contains a number of checks to reduce the incidence of missing and incorrect information. For instance, where feasible, new information is automatically validated against existing data, fields are made mandatory to force data entry and formats forced for particular fields. In addition, a series of reports are generated at regular intervals identifying what information is missing. Collection of this missing information is immediately sought to complete the record; hence few records include missing data.

For each subject, demographic information (gender, date of birth, date of death, postal code), diagnoses, caregiver relationship (for example, spouse, daughter, son, friend), primary reason for referral (pain, other symptom management, patient or family support, staff support, home consultation), location of death and program transition data were extracted from the PCP database. For each transition, the date, location of care (for example, home, acute care, long-term care) and an indicator of the services provided were included. These services could involve inpatient hospital care in the Palliative Care Unit, medical or surgical services from other acute inpatient care units, outpatient care provided by the Nova Scotia Cancer Center (NSCC) PCP clinic, PCP Home Support Service, care in a long-term care facility or care by a family physician.

Because individual level income information was not available from the PCP database, an aggregate measure of income derived from census information and grouped by enumeration area or 'neighbourhood' was employed. These 'neighbourhood income quintiles' were linked to the PCP database information via the postal code. Similarly, a geographical indicator of urban or rural residency, based on population density, was also generated and linked to the file. Prior to data linkage and release of the data for analysis, all personal identifying information was removed.

### Measures

We define a transition as either: 1) a change in location of where the patient was cared for by the PCP; or 2) a change in which service provided care. For example, a transition may be a move to or from the home, a specific acute care unit or a long-term care facility. A transition would also occur if the patient stayed in a single location, for example at home, but the care being provided is transferred from PCP staff (active care by PCP staff) to NSCC staff doctors (without PCP involvement), or to their family physician or home care nurse (non-active, no longer cared for by PCP staff) or vice versa. This change in service provider or 'transfer of care' transition scenario is illustrated below:

Home → Home

(Active: care by PCP staff) → (Non-active: care by family physician)

Each patient's diagnosis was categorized within one of six cancer groupings or as having problems other than cancer such as circulatory or respiratory disease. Site specific cancer groupings included the lung, colorectal, female breast, prostate, lymphatic and hematopietic tissue and all other cancers. Four locations of death were defined: in the hospital (excluding the inpatient PCP unit), within the inpatient PCP unit, at home and death occurring within a long-term care facility. Because the total time a patient was enrolled in the PCP varied, a 'survival' variable was created which was defined as the number of days between date of the initial admission to the PCP and death.

### Analysis

The total number and location of transitions experienced by patients from their initial admission to the PCP and during the final two and four weeks of life period were counted and summary statistics provided. Next, patient characteristics were examined, categorized and descriptive statistics presented.

Due to the highly skewed distribution of the total number of transitions experienced by patients admitted to the PCP, negative binomial regression was used to examine the association between total transitions and patient characteristics. To account for the variation in length of survival time, the time between each patient's date of enrollment and death, the log of survival was incorporated as an 'offset' in each regression model. Univariate or unadjusted regression analysis was followed by multivariate regression where patient characteristics found to be significantly associated with the total number of transitions, at the 0.05 level of significance in the unadjusted analysis were included in the initial model. Manual backwards elimination methods were then used to develop the most parsimonious model of total number of transitions and patient characteristics. All patient characteristics retained in the final multivariate model were significant at the 0.05 level. Regression coefficients are exponentiated and reported as rate ratios (RR). Similarly, the association between patient characteristics and the number of transitions occurring within the four and two week periods prior to death was examined. All analyses were conducted using SAS software [7].

## Results

Between January 1, 1998 and December 31, 2002, 3972 adult patients were admitted to the QEII PCP and had died on or prior to December 31, 2002. Just over half were male (52%), 65% were 65 years of age or older (mean 68.5 years, SD 13.7), the majority lived in an urban setting (70%) and 90% had been diagnosed with cancer. The major single cancer site was the lung accounting for 29% of all cancer diagnoses. The primary reason recorded for admission to the PCP was pain (46%), followed by the need for patient or family support (23%) and need for other symptom control (22%). Overall, just over half of patients admitted to the PCP (51%) experienced a hospital death outside the inpatient PCP unit whereas 31% died in their home. However, home deaths among PCP patients who had received at least one home visit by the PCP team, was found to be much higher, at 58.5%. Survival time, the number of days between program admission and death, varied from 0 days (died same day as admitted to the PCP) to 1688 days, with a mean of 100.6 (SD 163.2) and median of 45 days. Eighty five percent of patients admitted to the PCP survived 6 months or less. A table of relevant patient characteristics may be found in a previously published article [6].

In total, 5903 transitions were experienced by PCP patients over the five year study period (mean 1.5, SD 1.8; median 1; range 0 – 21). Twenty eight percent did not experience a transition following admission to the PCP, an indication that this group of patients experienced no change in the care provided or the location of care from the point of PCP admission to death. Over 40% experienced one transition while only 6.3% of PCP patients experienced five or more transitions. During the last four and two weeks of life, 47% and 36% of patients, respectively, experienced at least one transition. The mean number of transitions among patients during the last four weeks (mean 0.6, SD 0.8; median 0; range 0–6) and last two weeks (mean 0.4, SD 0.6; median 0; range 0–4) prior to death proved less than one. Further information pertaining to the number and type of transitions experienced by PCP patients are detailed as part of an alternate article by the authors [6].

Results from the examination of the association between patient characteristics and the total number of transitions experienced using regression techniques are illustrated in Table 1. After accounting for all other characteristics retained in the final multivariate model, fewer transitions were associated with being female (adjusted rate ratio [RR] 0.91; 95% confidence interval [CI] 0.84, 0.99), increasing age (for patients aged 85+ years compared to those <65 years, adjusted RR 0.81; 95%CI 0.69, 0.95), dying in a long term care facility compared to home (adjusted RR 0.78; 95%CI 0.62, 0.97) and over time (e.g. compared to 1998, for deaths in 2002, adjusted RR 0.73; 95%CI 0.65, 0.83). A greater number of transitions were associated with death in hospital, both in the PCP inpatient unit and other acute care units. Patients dying in the PCP inpatient unit experienced 118% more transitions than patients who died at home (adjusted RR 2.18; 95%CI 1.97, 2.42) whereas patients dying in other hospital acute care units experienced 19% more (adjusted RR 1.19; 95%CI 1.09, 1.31). Compared to patients who did not have a cancer diagnosis, a greater number of transitions were experienced by those dying with cancer of the lung (adjusted RR 1.29; 95%CI 1.06, 1.58), lymphatic and hematopoietic tissue cancers (adjusted RR 1.39; 95%CI 1.09, 1.77) and all other cancer sites combined (adjusted RR 1.22; 95%CI 1.01, 1.47). The number of transitions among patients with colorectal cancer, female breast cancer and prostate cancer did not differ significantly from patients who were dying from disease other than cancer. Although significantly associated with the total number of transitions in the unadjusted analysis, the relationship of the primary caregiver to the patient was no longer a significant factor following multivariate adjustments. Urban or rural residency, neighbourhood income quintile and the primary reason for referral to the PCP were not significantly associated with the total number of transitions from time of program admission to death, in the unadjusted or final multivariate regression models.

**Table 1:** The association of patient characteristics to the total number of transitions

**Characteristic**	**Rate Ratio (RR)**	
	
	**Unadjusted (95% CI)**	Adjusted* (95% CI)
**Gender **(vs male)		
Female	0.86 (0.80, 0.94)	0.91 (0.84, 0.99)

**Age **(vs <65 years)		
65–74	0.84 (0.76, 0.93)	0.86 (0.78, 0.95)
75–84	0.74 (0.67, 0.82)	0.82 (0.74, 0.90)
85+	0.69 (0.59, 0.81)	0.81 (0.69, 0.95)

**Location of death **(vs home)		
Inpatient PCP unit	2.17 (1.95, 2.42)	2.18 (1.97, 2.42)
Hospital	1.23 (1.12, 1.35)	1.19 (1.09, 1.31)
Long term care	0.70 (0.55, 0.88)	0.78 (0.62, 0.97)

**Year of death **(vs 1998)		
1999	0.81 (0.71, 0.93)	0.77 (0.68, 0.88)
2000	0.86 (0.76, 0.99)	0.77 (0.67, 0.87)
2001	0.75 (0.65, 0.86)	0.69 (0.60, 0.78)
2002	0.82 (0.71, 0.94)	0.73 (0.64, 0.83)

**Diagnoses **(vs other disease, no cancer)		
Lung cancer	1.54 (1.28, 1.86)	1.29 (1.06, 1.58)
Colorectal	1.26 (1.02, 1.55)	1.08 (0.88, 1.33)
Female breast	1.09 (0.86, 1.35)	0.91 (0.73, 1.14)
Prostate	1.12 (0.88, 1.43)	1.00 (0.79, 1.27)
Lymphatic & hematopoietic tissue	1.49 (1.16, 1.90)	1.39 (1.09, 1.77)
All other cancers	1.43 (1.19, 1.72)	1.22 (1.01, 1.47)

**Census residency indicator **(vs urban)		
Urban	0.95 (0.86, 1.03)	-

**Neighbourhood income quintile **(vs upper middle)		
Low	1.07 (0.94, 1.22)	-
Lower middle	1.04 (0.91, 1.19)	
Middle	1.01 (0.88, 1.14)	
Upper	1.04 (0.91, 1.18)	

**Caregiver relationship **(vs spouse/partner)		
Child	0.85 (0.77, 0.94)	-
Parents/other relations	1.08 (0.96, 1.22)	
Other	0.94 (0.74, 1.19)	

**Primary reason for referral to PCP **(vs home consultation)		
Pain	0.93 (0.84, 1.11)	-
Other symptom control	0.85 (0.73, 0.99)	
Patient or family support	0.90 (0.78, 1.05)	
Staff support	0.94 (0.71, 1.25)	

* Controlled for all other variables in the model. The census residency indicator, neighbourhood income quintile, relationship of the primary caregiver and the primary reason for referral to the PCP were not significantly associated with the total number of transitions in the final multivariate regression model.

Notes: 1. The log number of days between date of PCP admission and death for each patient was accounted for in the regression as the 'offset'.

2. The regression coefficients were exponentiated to express effects as rate ratios.

During the last four and two weeks of life, sex, diagnoses, location of death and year of death but not age, remained significantly associated with the number of transitions during the time period (Table 2). Also significantly associated with the total number of transitions during the two and four week periods prior to death was the primary reason for referral to the PCP. During these final weeks of life a greater number of transitions was associated with patients citing pain (at four weeks, adjusted RR 1.38; 95%CI 1.04, 1.82; at two weeks, adjusted RR 1.46; 95%CI 1.06, 2.01) and symptom control (at four weeks, adjusted RR 1.52; 95%CI 1.13, 2.06; at two weeks, adjusted RR 1.56; 95%CI 1.11, 2.20) as reasons for referral to the PCP compared to the need for a home consultation. As death drew nearer, patients who died in the PCP unit and those in other hospital wards tended to experience more transitions. For instance, during the four and two week period prior to death, patients dying in the PCP unit had more transitions than those who died at home (within four weeks prior to death, adjusted RR 2.98; 95%CI 2.45, 3.62; within two weeks prior to death, adjusted RR 4.09; 95%CI 3.26, 5.13). During these final weeks of life patients diagnosed with female breast (adjusted RR 0.53; 95%CI 0.34, 0.82), prostate (adjusted RR 0.38; 95%CI 0.23, 0.61) and colorectal cancers (adjusted RR 0.66; 95%CI 0.44, 0.99) were all found to experience fewer transitions than PCP patients who did not have a cancer diagnosis. This result differs from that estimated during the time period from initial PCP admission to death where no differences were found. In contrast, although experiencing significantly more transitions in the period spanning from initial PCP admission to death, patients with cancer of the lung, lymphatic and hematopoietic tissue and all other sites experienced a similar number of transitions as those with other disease during the final four and two weeks of life.

**Table 2:** The association of patient characteristics to the total number of transitions during the last two and four weeks of life

**Characteristic**	Adjusted* **Rate Ratio (RR) (95% CI)**	
	
	**Four weeks prior to death**	**Two weeks prior to death**
**Gender **(vs male)		
Female	0.77 (0.66, 0.89)	0.69 (0.58, 0.82)

**Location of death **(vs home)		
Inpatient PCP unit	2.98 (2.45, 3.62)	4.09 (3.26, 5.13)
Hospital	1.46 (1.23, 1.72)	1.94 (1.59, 2.37)
Long term care	0.39 (0.24, 0.64)	0.54 (0.31, 0.92)

**Year of death **(vs 1998)		
1999	0.60 (0.48, 0.76)	0.54 (0.41, 0.70)
2000	0.72 (0.57, 0.90)	0.69 (0.53, 0.89)
2001	0.58 (0.46, 0.73)	0.57 (0.44, 0.75)
2002	0.69 (0.54, 0.87)	0.60 (0.46, 0.79)

**Diagnoses **(vs other disease, no cancer)		
Lung cancer	0.97 (0.71, 1.33)	0.87 (0.61, 1.25)
Colorectal	0.68 (0.48, 0.97)	0.66 (0.44, 0.99)
Female breast	0.57 (0.39, 0.84)	0.53 (0.34, 0.82)
Prostate	0.45 (0.29, 0.69)	0.38 (0.23, 0.61)
Lymphatic & hematopoietic tissue	1.08 (0.72, 1.62)	1.01 (0.63, 1.62)
All other cancers	0.89 (0.65, 1.21)	0.75 (0.52, 1.06)

**Primary reason for referral to PCP **(vs home consultation)		
Pain	1.38 (1.04, 1.82)	1.46 (1.06, 2.01)
Other symptom control	1.52 (1.13, 2.06)	1.56 (1.11, 2.20)
Patient or family support	1.21 (0.90, 1.62)	1.18 (0.84, 1.66)
Staff support	1.49 (0.90, 2.49)	1.26 (0.69, 2.28)

* Controlled for all other variables in model. Age, the census residency indicator, neighbourhood income quintile and relationship of the primary caregiver were not significantly associated with the total number of transitions in the final multivariate regression model.

Notes: 1. The log number of days between date of PCP admission and death for each patient was accounted for in the regression as the 'offset'.

2. The regression coefficients were exponentiated to express effects as rate ratios.

Table 3 provides a summary of patient characteristics found to be significantly associated with total transitions within each time period prior to death.

**Table 3:** Summary of patient characteristics found to be significantly associated with total transitions by time period prior to death

	Association of predictor to total transitions*		
	Time periods when transitions experienced		
	
	PCP registration to death	Four weeks prior to death	Two weeks prior to death

**Total transitions experienced**(Mean; Standard Deviation [SD])	5903 (1.5, SD 1.8)	2446 (0.6, SD 0.8)	1654 (0.4, SD 0.6)

**Effect of patient characteristic on total transitions**			

**Gender **(vs male)			
Female gender associated with fewer transitions			

**Age **(vs < 65 years)			
Older age associated with fewer transitions			

**Location of death **(vs home)			
Death occurring in the inpatient PCP unit or acute hospital setting associated with more transitions			
Death occurring in a long term care facility associated with fewer transitions			

**Year of death **(vs 1998)			
Patients dying between 1999 and 2002 associated with fewer transitions			

**Diagnoses **(vs other disease, no cancer)			
A cancer diagnosis associated with more transitions			
A cancer diagnosis associated with fewer transitions			

**Primary reason for referral to PCP **(vs home consultation)			
Referral for pain or other symptom control associated with more transitions			

*  Multivariate regression analyses indicate a significant association at the 0.05 level of association

## Discussion

Overall, results suggest PCP patients who experienced a hospital death and those with a cancer diagnosis were more likely to undergo multiple transitions during the time period from initial PCP admission to death. Fewer transitions were associated with being female, elderly and having died more recently. In focusing on the last month of life, these associations change somewhat with age no longer being associated with the total number of transitions, some cancer diagnoses being similar to or related to fewer transitions than a non-cancer diagnosis, and pain and symptom control becoming a significant factor associated with a greater number of transitions.

During the time period from initial PCP admission to death, patients dying in the PCP inpatient unit were found to have twice as many transitions compared to those who died at home. Further, the number of transitions during the last two weeks of life was found to be four times higher among patients with a PCP inpatient death compared to a home death. Anecdotal evidence gained from clinical experience suggests patients admitted to the PCP inpatient unit have more 'complicated' needs with respect to pain, symptom control and psychosocial issues. Because of this, they tend to be readmitted to the unit following transitions elsewhere. Our data provide some evidence to support this view. During the last four and two weeks of life, pain and other symptom control as reasons for PCP referral were found to be significant factors associated with greater total transitions suggesting patients in the PCP inpatient unit and other acute care hospital wards are more in need of this form of care. However, even after adjustments are made for these pain and symptom factors, a clear association remains between having had multiple transitions and dying in the PCP inpatient unit. We suggest that once a relationship and a comfort level between patient and the PCP unit staff becomes established, the patient is more inclined to return to the PCP unit as death draws near.

Over the full time period from PCP admission to death (Table 1), patients referred to the PCP with cancer experienced significantly more transitions than the subset of referred patients without a cancer diagnosis. However, as death drew near (Table 2, last four and two weeks of life) this situation reversed and cancer patients experienced fewer transitions than non cancer patients. In the early phase of the move toward palliating cancer patients, oncological therapies and potential complications may necessitate more transitions in care. In general, cancer has a longer time course with a much slower and predictable decline allowing services to be organized more easily in the longer timeline available. Cancer patients often have clearer goals of care and end points. However, non cancer patients have more uncertainty of when they will die. Because there may be reversible mechanisms causing their decline and potential treatments available, the time when palliation is needed may be more difficult to define. A good example of this is congestive heart failure.

Women admitted to the PCP experienced fewer transitions than men during their total time in the program following adjustments for the effects of age, diagnosis, year of death and location of death. A reduced number of transitions among women is even more apparent during the last four and two weeks of life. Why would women make fewer transitions? Do women have different goals in where and how they are cared for during the end of life? Is a home death more desirable among women or does their disease progression and need for support differ from men? Closer examination of our data indicates a small, statistically insignificant difference in the number of home deaths among men and women who had been provided care at home at least once while a patient of the PCP (for women 51.4%; men 48.6%). Previous studies we conducted demonstrate that women with breast cancer are more likely to die out of hospital than women with other cancers and men in general [8]. An alternate view might also consider fewer transitions the result of women's needs during the end of life being overlooked by the health care system, in that they are not getting access to needed hospital or community services. Finally, we wonder if caregiver availability and ability to women might be different than for men? Each of these questions deserves further study in order to understand whether or not women have different needs necessitating fewer transitions or are being denied access to some elements of care.

Age was also a factor in the number of transitions a PCP patient may experience. After accounting for gender, diagnoses, year of death and location of death, fewer transitions were evident with increasing age. This association was evident only during the full time period from program admission to death and not a significant indicator during the last four or two weeks of life. Is this reflective of new developments in palliative care and chemotherapy regimens being given among younger patients, particularly when they are first admitted to the PCP? There is evidence that the elderly have less access to screening and state of the art treatment for cancer [9-11]. Or do younger patients have more aggressive disease demanding greater symptom control? Alternatively, it may be that older patients are already in hospital, home or long term care and inclined to stay in that situation.

Compared to the initial study year, 1998, the number of transitions among PCP patients in subsequent years has declined significantly. This reduction could be a reflection of more community services being available such as the expansion of home care [12] allowing people to stay at home when they might have otherwise needed hospitalization. The countering argument is that fewer hospital supports, most notably fewer acute hospital beds, are available in the more recent years of the study perhaps making transition to hospital less likely [13].

Although the number of beds available in the inpatient unit of the PCP has remained stable since 1997, the reduction in the number of transitions made by patients from 1999 onward may be a reflection of an increase in the number of Home Consult Registered Nurses (RN) associated with the program. In 1999 the number of home consult RNs rose from three to five.

The demographics of the adult patients admitted to the QEII Health Sciences Center PCP have changed slightly since program initiation in 1988 [3]. Compared to the 1988 to 1993 period, the number of males involved in the program declined by only 2% (from 54% in 1988–1993 to 52% 1998–2002), while the median number of survival days, the time from admission to the PCP to death, decreased from 54 days in 1988–1993 to 45 days in 1998–2002. This latter change reduces the number of survival days available to patients for transitions and demonstrates the need for our "survival time" adjustment. A reduction in the median number of days in the PCP may be an indication of less time available to affect planned, coordinated and integrated care in a way that might minimize transitions for patients. Alternatively, the reduction may be a reflection of changes in oncological practice for palliative regimes over the time period.

Although slightly more non cancer patients were admitted to the PCP in later years, cancer patients continue to constitute the majority of PCP admissions, in particular patients with cancer of the lung. Cancer of the lung persists as the most common site of cancer mortality in Canada with deaths in Nova Scotia being higher than the national average [14]. It is therefore reasonable to expect a greater percentage of lung cancer patients being admitted to the PCP. Our lung cancer patients represented 26% of all PCP patients, a slightly higher proportion than that reported in Ontario (20%) and United Kingdom (23%) palliative care settings [15,16]. Even though the proportion of elderly (≥ 75 years) patients in the program has increased over time from 28% (1993–1994) to 37% in the current study years (1998–2002), PCP involvement among patients less than 65 years of age remains relatively higher given that among all cancer deaths in Nova Scotia, 46% are among those 75 years or older and only 26% less than 65 years of age [8]. Possible under representation of the elderly in a PCP program has been reported by others and in our previous work [3,4,16]. This continues to raise concerns for us about where the elderly access specialized palliative care. Do they obtain palliative care from other ambulatory or home based care programs of long term care or other facilities or are they not getting the services they need [17]?

### Limitations

One of the major limitations to this study is the potential loss of transition information when a patient is no longer actively followed by the program. This may occur if a patient changes residence and no longer resides in the region served by the QEII PCP, their health had improved such that they are no longer considered palliative or they had a change in their personal caregiver. To complicate the problem, patients may return to the program at any point during their end of life. For all patients our database retains a record of their admission, death date and transitions made while in the program. It is therefore probable that inclusion of patients while not being actively followed for even a short period of time, could result in a conservative underestimation of the number of transitions made. Unfortunately, demographic information collected is somewhat limited. It would be helpful to have a much better description of the available supports to patients in the community such as whether a caregiver lives in the home, is available 24 hours a day, how many caregivers are available, what private insurance is available, etc. Having better measures of symptom severity would permit a more thorough analysis of the specific nature of symptoms that are associated with a greater number of transitions. To further pursue the study of factors associated with transitions during the end of life it may be necessary to design a study where additional information is collected prospectively.

## Conclusion

Many patients experience transitions or changes in location or care at the end of life. Our data suggest there is some variation in the number of these transitions associated with the demographics and diagnoses of patients. A number of issues need further exploration such as the associations with gender and age and how caregiver supports and symptom issues contribute to these. Also important is the need to facilitate continuity of care across settings since many transitions are inevitable and often integral to care.

## Competing interests

The author(s) declare they have no competing interests.

## Authors' contributions

BL and FB participated in the conceptualization and design of the project, the analysis and interpretation of the data, first-drafted the majority of the article, and incorporated co-authors' comments into the final draft. PC participated in the conceptualization and design of the project, interpretation of data, and revising of the manuscript. PM aided in the interpretation of the data and reviewed each draft for critical content. All authors gave approval to the final version.

## Pre-publication history

The pre-publication history for this paper can be accessed here:


